# BM-MSCs alleviate diabetic nephropathy in male rats by regulating ER stress, oxidative stress, inflammation, and apoptotic pathways

**DOI:** 10.3389/fphar.2023.1265230

**Published:** 2023-11-16

**Authors:** Tarek Khamis, Adel Abdelkhalek, Hussein Abdellatif, Nourelden Dwidar, Ahmed Said, Rama Ahmed, Kerolos Wagdy, Rowina Elgarhy, Rawan Eltahan, Hisham Mohamed, Eman Said Amer, Maria Hanna, Tarek Ragab, Abdallah Kishk, Judy Wael, Eyad Sarhan, Linda Saweres, Mohamed Reda, Sara Elkomy, Abdalah Mohamed, Abdullah Samy, Ateya Khafaga, Youliana Shaker, Hamdy Yehia, Asma Alanazi, Mohammed Alassiri, Emil Tîrziu, Iulia Maria Bucur, Ahmed Hamed Arisha

**Affiliations:** ^1^ Department of Pharmacology and Laboratory of Biotechnology, Faculty of Veterinary Medicine, Zagazig University, Zagazig, Egypt; ^2^ Faculty of Veterinary Medicine, Badr University in Cairo, Badr, Egypt; ^3^ Department of Human and Clinical Anatomy, College of Medicine and Health Sciences, Sultan Qaboos University, Muscat, Oman; ^4^ Anatomy and Embryology Department, Faculty of Medicine, Mansoura University, Mansoura, Egypt; ^5^ College of Medicine, King Saud Bin Abdulaziz University for Health Sciences (KSAU-HS), Riyadh, Saudi Arabia; ^6^ King Abdullah International Medical Research Center, Riyadh, Saudi Arabia; ^7^ Department of Basic Sciences, College of Science and Health Professions, King Saud Bin Abdulaziz University for Health Sciences (KSAU-HS), Riyadh, Saudi Arabia; ^8^ Department of Pathology and Laboratory Medicine, King Abdulaziz Medical City (KAMC), Ministry of the National Guard—Health Affairs, Riyadh, Saudi Arabia; ^9^ Department of Animal Production and Veterinary Public Health, Faculty of Veterinary Medicine, University of Life Sciences, “King Mihai I” from Timisoara [ULST], Timisoara, Romania; ^10^ Department of Animal Physiology and Biochemistry, Faculty of Veterinary Medicine, Badr University in Cairo, Badr, Egypt; ^11^ Department of Physiology, Laboratory of Biotechnology, Faculty of Veterinary Medicine, Zagazig University, Zagazig, Egypt

**Keywords:** diabetic nephropathy, mesenchymal stem cells, bone marrow-derived mesenchymal stem cells, diabetes, apoptosis, ER stress, inflammation, intermediate filament proteins

## Abstract

**Introduction:** Diabetic nephropathy (DN), a chronic kidney disease, is a major cause of end-stage kidney disease worldwide. Mesenchymal stem cells (MSCs) have become a promising option to mitigate several diabetic complications.

**Methods:** In this study, we evaluated the therapeutic potential of bone marrow-derived mesenchymal stem cells (BM-MSCs) in a rat model of STZ-induced DN. After the confirmation of diabetes, rats were treated with BM-MSCs and sacrificed at week 12 after treatment.

**Results:** Our results showed that STZ-induced DN rats had extensive histopathological changes, significant upregulation in mRNA expression of renal apoptotic markers, ER stress markers, inflammatory markers, fibronectin, and intermediate filament proteins, and reduction of positive immunostaining of PCNA and elevated P53 in kidney tissue compared to the control group. BM-MSC therapy significantly improved renal histopathological changes, reduced renal apoptosis, ER stress, inflammation, and intermediate filament proteins, as well as increased positive immunostaining of PCNA and reduced P53 in renal tissue compared to the STZ-induced DN group.

**Conclusion:** In conclusion, our study indicates that BM-MSCs may have therapeutic potential for the treatment of DN and provide important insights into their potential use as a novel therapeutic approach for DN.

## 1 Introduction

Diabetic nephropathy (DN) is a frequent diabetic consequence and a primary cause of end-stage renal failure. The pathogenesis of DN is complex and includes several pathways/mechanisms, such as hyperglycemia, oxidative stress, apoptosis, inflammation, endoplasmic reticulum (ER) stress, and fibrosis (Zhuang et al., 2019). DN causes progressive kidney impairment, proteinuria, and reduced renal function, eventually leading to end-stage renal disease (ESRD). DN is accompanied by damage to podocytes that are involved in cellular hypertrophy, podocytopenia, and glomerulosclerosis (Liu and Tang, 2016). Unlike other types of cells, podocytes have a limited renewal capability once damaged, and the glomerular filtration barrier becomes leaky, resulting in proteinuria and worsening podocyte destruction (Mathieson, 2012). Oxidative stress becomes apparent when there is an imbalance between the generation of reactive oxygen species (ROS) and body’s ability to neutralize them with antioxidants. Hyperglycemia, advanced glycation end products (AGEs), and dyslipidemia all contribute to oxidative stress in DN. Previous studies (Lee et al., 2003; Xu et al., 2017; Amaral et al., 2018; Gong et al., 2019) reported that hyperglycemia-induced renal oxidative stress via decreasing mitochondrial membrane potential increases the generation of ROS and RNS that caused micro- and macrovascular alteration ended with DNA damage, the overexpression of extracellular matrix protein precipitation, mesangial expansion, glomerular fibrosis, and glomerular atrophy (Kashihara et al., 2010). Oxidative stress can also cause the release of proinflammatory cytokines and chemokines, which can lead to inflammation and additional kidney damage. Furthermore, oxidative stress can induce apoptosis and autophagy, resulting in renal cellular death.

The ER is a cellular organelle that regulates protein folding and quality. ER stress occurs when the ER capacity to fold and process proteins is exceeded, resulting in a buildup of misfolded or unfolded proteins. Hyperglycemia-induced oxidative stress decreases sarco/endoplasmic reticulum Ca +2 ATPase, which in turn decreases ER Ca +2 content needed for proper protein folding, which leads to the propagation of unfolded and misfolded proteins, translocation of ER chaperone glucose-regulated protein 78 (GRP78) and GRP96 to the cytoplasmic membrane, induced ER-mediated cytokine secretions, and apoptosis (Clark and Urano, 2016). On the same line, other studies reported that diabetic nephropathy significantly upregulated the endoplasmic reticulum stress markers: ATF3, ATF6, ATF4, JNK, CHOP, BIP, and XBP1(Wang Z et al., 2017; Xu et al., 2017; Yang and Wu, 2018; Shibusawa et al., 2019). In addition, renal ER stress activation may be attributed to hyperglycemia, free fatty acids (FFA), and AGE, which led to ER Ca+2 depletion, which exacerbated ER stress and apoptosis, activating exogenous chaperones (Morse et al., 2010; Cao et al., 2014) to reestablish cellular homeostasis. However, current therapeutic strategies that target hyperglycemia (via glucose-lowering agents) and hypertension (via renin–angiotensin–aldosterone system (RAAS) inhibitors) do not provide adequate control or reversal of diabetic nephropathy (Ahmad, 2015). Although those strategies aim to control hyperglycemia and target hemodynamic changes to slow down the progression of kidney injury, it does not include any therapeutic intervention to treat the damaged renal cells (Ahmad, 2015).

Stem cells (SCs), characterized by self-renewal and plasticity (Wagers and Weissman, 2004), can be classified into embryonic stem cells (ESCs) and adult stem cells (ASCs) (Bissels et al., 2016). ASCs are multipotent cells with immunomodulatory and regenerative characteristics that can develop into various cell types. ASCs of various kinds, including mesenchymal stem cells (MSCs), olfactory, endothelial stem cells (ESCs), neural stem cells, and hematopoietic stem cells (HSCs) (Bongso and Lee, 2005), can be used in cell-based treatment for liver cirrhosis (Mu et al., 2018), spinal cord injury (Hakim et al., 2019), and peripheral vascular disease (Yan et al., 2013). ASCs’ effective homing and differentiability may restrict their therapeutic and clinical uses (Stonesifer et al., 2017). MSCs are an excellent therapeutic option due to their anti-inflammatory (Kim et al., 2015), immunomodulatory (Kassis et al., 2008), and antiapoptotic (Wang et al., 2017) properties. MSCs might also reduce oxidative stress (Francois et al., 2013; Sherif et al., 2018) and release trophic factors including hepatocyte growth factor (HGF) and vascular endothelial growth factor (VEGF) (Kaingade et al., 2016). Each of the types of MSCs has distinct characteristics to consider when used in cell-based therapy. Bone marrow-derived MSCs (BM-MSCs) are the most frequently utilized form of MSCs. BM-MSCs may self-renew and differentiate into a variety of cell types. It is crucial to highlight that stem cell-based therapy is still a developing field, and more research is needed to better understand the mechanisms of action of stem cells in disease models such as DN and to maximize their therapeutic potential. As a result, the current work was aimed at investigating into the potential regenerative properties of BM-MSCs in a diabetic nephropathy rat model.

## 2 Materials and methods

### 2.1 Experimental design

Forty-five mature male Sprague Dawley (SD) rats were divided into three groups of 15 rats in each group: G1, the control group; G2; and G3. The G2 diabetic group received a single intraperitoneal injection of STZ 65 mg/kg (Furman, 2015; Khamis et al., 2020; Khamis et al., 2021). G3 diabetic group rats were administered intraperitoneally once with BM-MSCs 2 × 10^7^ 4 weeks after diabetes was induced. After the 4th and 12th weeks of treatment, rats were placed in individual metabolic cages for 24 h to collect urine samples using sterile containers. Urine samples were centrifuged for 15 min at 1,000 g to remove any particulates and stored at -20°C until analysis. Glomerular filtration was assessed by creatinine clearance based on serum and urine creatinine levels, with values expressed in mL/min, computed with the following formula: Clcr = urine creatinine (mg/dL) × urine flow (mL/min)/serum creatinine (mg/dL). Urine flow was calculated dividing 24 h of urine volume by 1,440, which corresponds to the number of minutes in 24 h (60 min × 24 h = 1,440): urine flow (mL/min) = value of urine volume (24 h)/1,440.

Twelve weeks after STZ injection, two blood samples were collected from each rat via medial eye canthus: one sample with sodium fluoride for measuring blood glucose levels and another sample without anticoagulants for serum hormonal assays. The volume of withdrawn blood was variable, and on average, 1 mL was collected at week 4, while 3 mL were collected at week 12. Rats were euthanized, and tissue samples were obtained. The serum was collected and maintained at −80°C for later study. The kidney tissues were quickly removed and split into two portions, the first of which was preserved on neutral buffered formalin (NBF-10%) for histological and immunohistochemical study, and the second was 50 mg collected using 1 mL QIAzol (QIAGEN, Germany) for total RNA extraction and stored at -80°C for further use.

### 2.2 MSC isolation, identification, and administration of BM-MSCs

According to the protocol of Smajilagić et al. (2013), 12-week-old rats were sacrificed, and their femur and tibia were aseptically extracted following the sacrifice procedure. The cancellous bone was extracted from the femur and tibia specimens and subjected to 3–5 washes using 1x phosphate-buffered saline (PBS) obtained from Lonza Bioscience. Subsequently, the bone marrows were rinsed using Dulbecco’s modified Eagle medium (DMEM) culture media (Lonza Bioscience) containing 10% (V/V) FBS (Lonza Bioscience) and 50 IU L-1 penicillin, 2 mM L-glutamine, and 50 µg mL-1 streptomycin (Lonza Bioscience). The released cells were collected and placed in a culture flask with a surface area of 75 cm^2^. The flask contained 15 mL of DMEM culture media from Lonza Bioscience. The cell cultures were incubated at 37°C in a controlled environment with a humidity level of 95% air and 5% carbon dioxide. The cells were left to adhere for 3 days. Following this, the non-adherent cell population was eliminated, and the culture medium was substituted with new culture DMEM supplemented with 10% (V/V) fetal bovine serum (FBS), 50 IU L^-1^ penicillin, 2 mM L-glutamine, and 50 µg mL-1 streptomycin (Lonza Bioscience). The culture media underwent biweekly changes. Following the initial passage, the adhering cells were dissociated using a solution of 0.25% trypsin-EDTA (Lonza Bioscience) and subsequently cultivated for three consecutive passages. After the third passage, the cells were identified by flow cytometrical analysis of CD45, CD34, CD90, CD73, CD44, CD14, and CD105 (Becton Dickinson, San Diego, CA, United States) (Khamis et al., 2020). Before BM-MSC transplantation, cells were labeled with the PKH-26 cell linker (Sigma, Aldrich) to track the homing of the transplanted cells in the renal tissues following the supplier guidelines.

### 2.3 Blood and urine biochemical analysis

The fasting blood glucose was monitored every 2 weeks (Khamis et al., 2021). Rat albumin and microalbumin were measured according to Oraby et al. (2019). Urine and blood urea were determined according to the method previously described (Pöge and Kothe, 1983). Urine and serum creatinine were measured according to the method previously described (Young and Friedman, 2001).

### 2.4 qRT-PCR analysis

Total RNA was extracted from 30 mg of kidney tissue using QIAzol (QIAGEN, Germany), and cDNA was synthesized using a High-Capacity cDNA Reverse Transcription Kit (Applied Biosystems, United States) (Khamis et al., 2020; Khamis et al., 2021). Real-time RT-PCR was carried out using a Rotor-Gene Q 2 plex real-time PCR system (QIAGEN, Germany) using TOPreal^TM^ qPCR 2X PreMIX (Enzynomics, Korea) and primers listed in Supplementary Table S1. The target gene results were normalized by the mRNA expression of GAPDH and expressed as fold changes relative to the control group using the 2^−ΔΔCT^ method (Livak and Schmittgen, 2001).

### 2.5 Histopathological examination

The paraffin wax blocks containing the collected renal tissue were cut into 5 μm-thick sections that were stained using hematoxylin and eosin (H&E) or Masson’s trichrome (MTC) (Layton and Suvarna, 2013).

### 2.6 Immunohistochemical analysis

The avidin–biotin–peroxidase complex technique for the immunohistochemical staining of P53 and PCNA in the kidney was used. In brief, formalin-fixed sections were heated for 20 min in a 10 mM citrate buffer (pH 6.0), cooled for 20 min, and then, subjected at 4°C to primary antibodies overnight. PBS was used for 2X washing of the stained section with the primary antibody for 5 min; then, sections were successively treated for 15 min with peroxidase-conjugated streptavidin (1:3,000 in PBS) and the appropriate secondary antibody. Visualization of immunolabeling was performed using 0.02% 3,3- diaminobenzidine tetrahydrochloride. The counterstained hematoxylin slices were dried using gradient ethanol and mounted in Canada balsam. For the morphometric study, ImageJ (National Institutes of Health, United States) was used.

### 2.7 Statistical analysis

Mean ± standard error mean (SEM) was used to describe continuous variables. In homogeneous data, one-way analysis of variance (ANOVA) was used, followed by post-hoc Tukey’s test for multiple group comparison using SPSS (Chicago, IL, United States); a *p*-value lower than 0.05 was considered statistically significant (Stehlik-Barry and Babinec, 2017).

## 3 Results

### 3.1 BM-MSC isolation, identification, and renal homing

**FIGURE 1:**
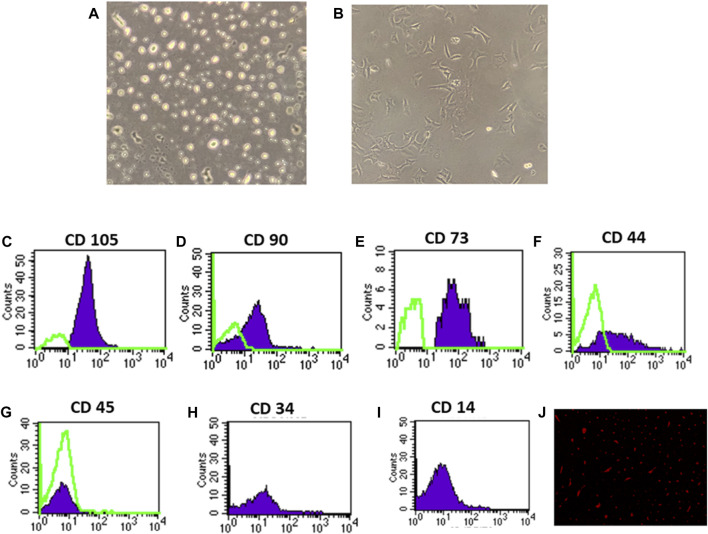
Identification and homing of BM-MSCs in diabetic rat renal tissue **(A–J)**. **(A)** BM-MSC isolation on the third day of culture; **(B)** BM-MSC isolation on the seventh day of culture **(C–I)**. Flow cytometric detection of BM-MSCs: **(C)** BM-MSC cell populations were +ve for CD105; **(D)** BM-MSCS cell populations were +ve for CD90; **(E)** BM-MSC cell populations were +ve for CD73; **(F)** BM-MSC cell populations were +ve for CD44; **(G)** BM-MSC cell populations were -ve for CD45; and **(H)** BM-MSC cell populations were -ve for CD34. **(I)** BM-MSC cell populations were -ve for CD14, and **(J)** PKH26 was used to identify BM-MSC homing in renal tissue.

On the third day of culture, isolated MSCs from rat bone marrow were able to adhere to the bottom of the culture flask (Figures 1A, B). These isolated cells’ nuclei were either elongated or rounded (Figure 1A). The isolated cells had a characteristic fibroblastic appearance after 7 days of culture (Figure 1B). Flow cytometric analysis showed that the separated cell populations were CD105, CD90, CD73, and CD44 positive (Figures 1C, F). It was also negative for the hematopoietic stem cell markers CD45, CD34, and CD14 (Figures 1G, I). The findings revealed that the isolated cells often exhibit the features of MSCs. Meanwhile, fluorescence microscopic examination of the BM-MSC-treated diabetic rats revealed a red fluorescent color of PKH-26-positive BM-MSCs dispersed with the renal tissue (Figure 1J).

### 3.2 Effect of BM-MSC transplantation on renal function tests

**FIGURE 2:**
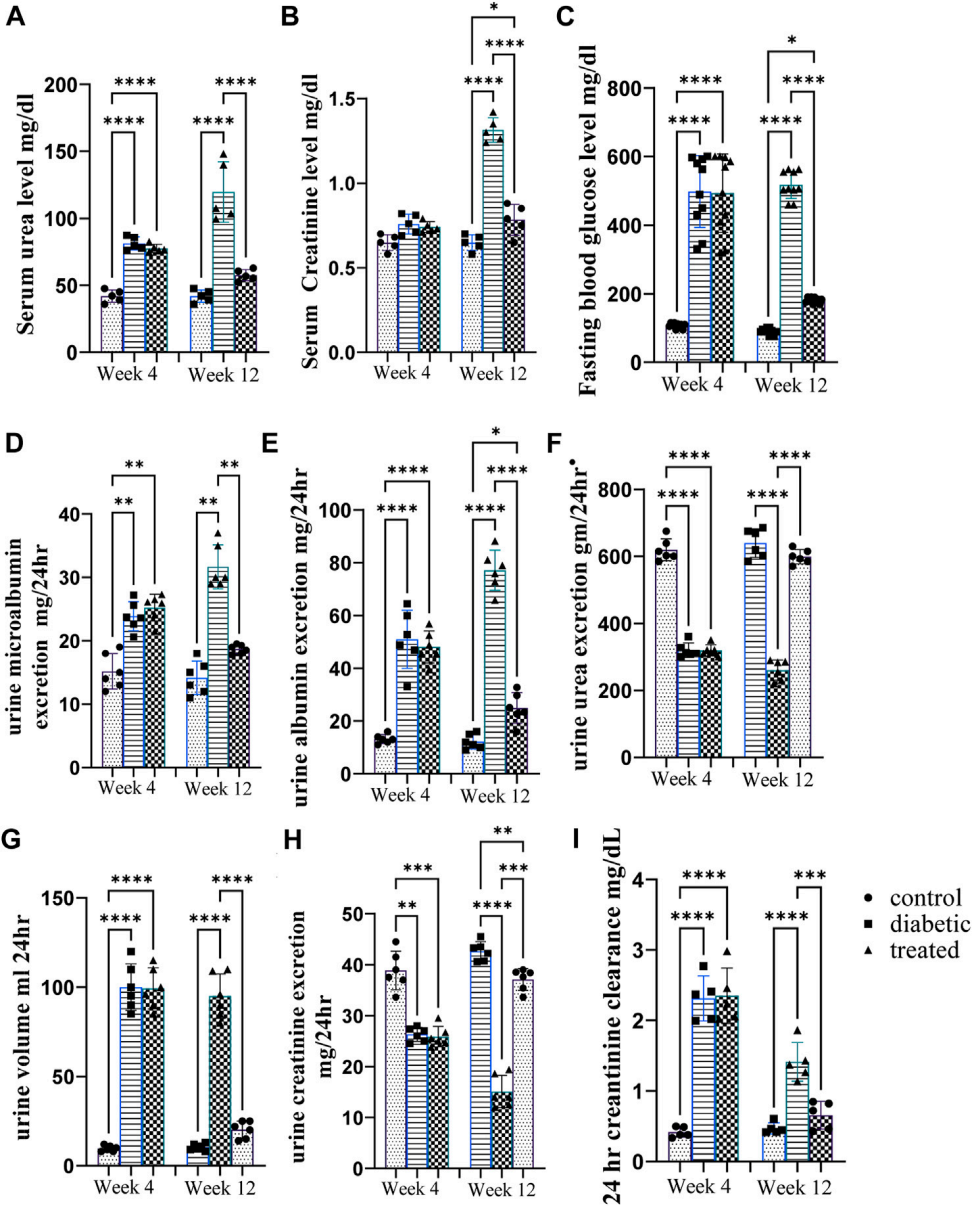
Effect of BM-MSC administration on renal function tests at the 4thand 12th weeks after STZ induction of diabetes **(A–H)**. **(A)** Serum urea level mg/dL, **(B)** serum creatinine level mg/dL, **(C)** mean value of FBG level mg/dL, **(D)** urine microalbumin excretion mg/24 h, **(E)** urine albumin excretion mg/24 h, **(F)** urine urea excretion gm/24 h, **(G)** urine volume/mL per 24 h, **(H)** urine creatinine excretion mg/24 h, and **(I)** 24 h creatinine clearance mg/dL. Values represent the mean ± SEM of 10 rats per group. *p < 0.05, **p < 0.01, ***p < 0.001, and ****p < 0.0001.

Type 1 diabetes induction caused a significant increase in the mean urine volume, serum urea level, serum creatinine level, urine microalbumin, 24 h creatinine clearance, and albumin excretion and a significant decrease in urine urea excretion and creatinine excretion at the 12th week as well as 4th week after diabetes induction in diabetic rats compared with control rats (Figures 2A-J). However, intraperitoneal BM-MSC administration elicited a significant decrease in the mean value of urine volume, serum urea level, serum creatinine level, urine microalbumin, 24 h creatinine clearance, and albumin excretion and a significant increase in urine urea excretion and creatinine excretion at the 12th week as well as 4th week after diabetes induction in diabetic rats compared with control group rats (Figures 2A-I).

### 3.3 Effect of BM-MSC transplantation on the expression of renal intermediate filament proteins

**FIGURE 3:**
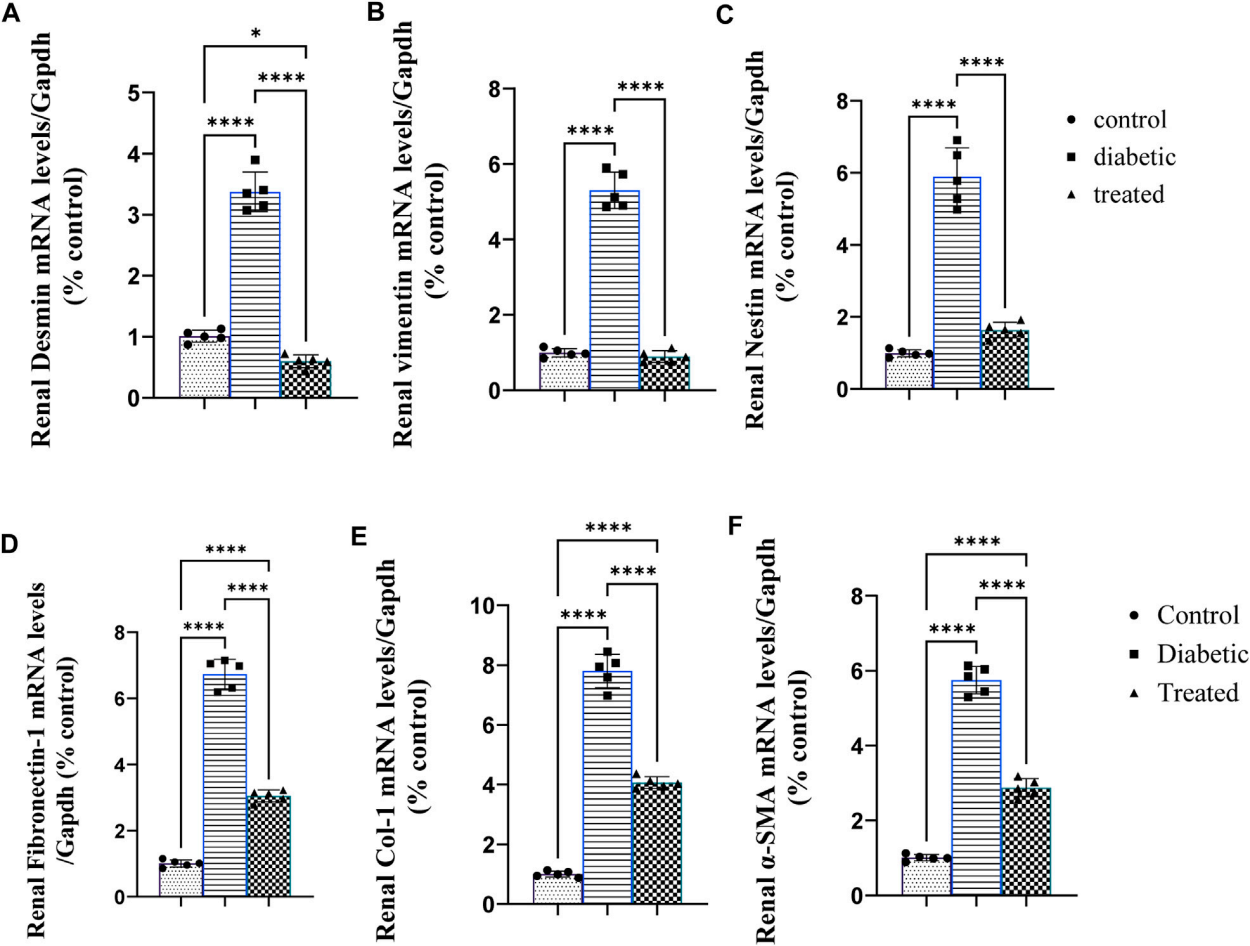
Effect of BM-MSC administration on the mRNA expression of renal intermediate filament protein genes **(A–F)**: **(A)** renal desmin, **(B)** renal vimentin, **(C)** renal nestin, **(D)** renal fibronectin-1, **(E)** renal Coli-1, and **(F)** renalalpha-smooth muscle actin (α-SMA). Values represent the mean ± SEM of 10 rats per group. *p < 0.05, **p < 0.01, ***p < 0.001, and ****p < 0.0001.

Type 1 diabetes induced a significant upregulation in the mean fold change of relative mRNA expression of the renal cytoskeleton (stress indicators): desmin, vimentin, nestin, fibronectin-1, Coli-1, and α-SMA (Figures 3A–F). On the contrary, BM-MSC administration significantly downregulated the mean fold change of the relative mRNA expression of the renal cytoskeleton (stress indicators): desmin, vimentin, nestin, fibronectin-1, Coli-1, and α-SMA (Figures 3A–F).

### 3.4 Effects of BM-MSC transplantation on the expression of renal inflammatory markers

**FIGURE 4:**
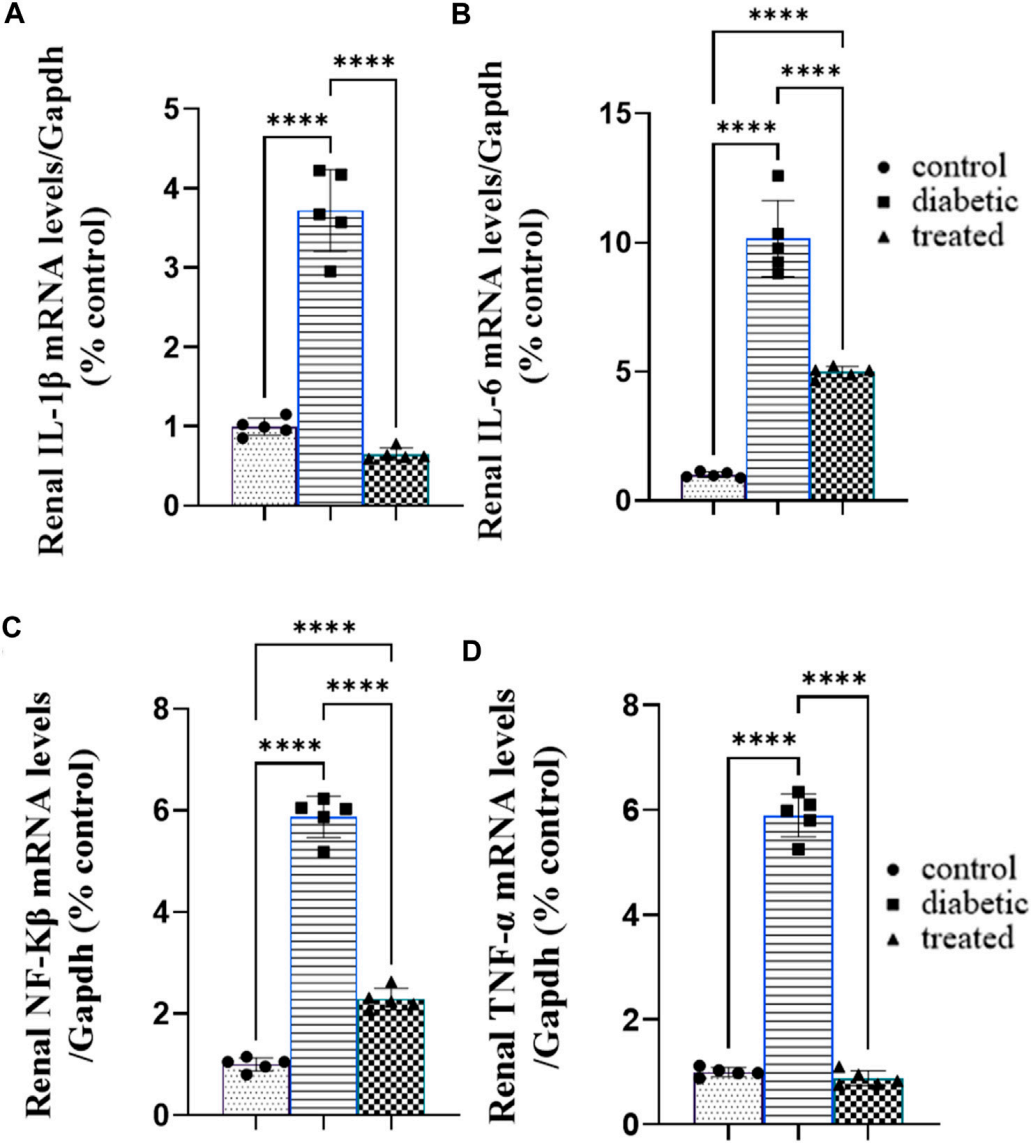
Effect of BM-MSC administration on the mRNA expression of renal proinflammatory markers **(A–D)**: **(A)** renal IL-1β, **(B)** renal IL-6, **(C)** renal NFKβ, and **(D)** renal TNFα. Values represent the mean ± SEM of 10 rats per group. *p < 0.05, **p < 0.01, ***p < 0.001, and ****p < 0.0001.

Type 1 diabetes caused a significant increase in the mean fold change in the relative mRNA expression of the renal proinflammatory markers such as nuclear factor-κB NFKβ, tumor necrosis factor (TNFα), interleukin-1β (IL-1β), and interleukin-6 (IL-6) compared with the control group (Figures 4A–D). On the contrary, BM-MSC administration significantly downregulated the mean fold change of the relative mRNA expression of the renal proinflammatory markers such as NFKβ, IL1β, TNFα, and IL6 compared to the diabetic group (Figures 3A–D).

### 3.5 Effect of BM-MSC transplantation on the mRNA expression of renal ER stress

**FIGURE 5:**
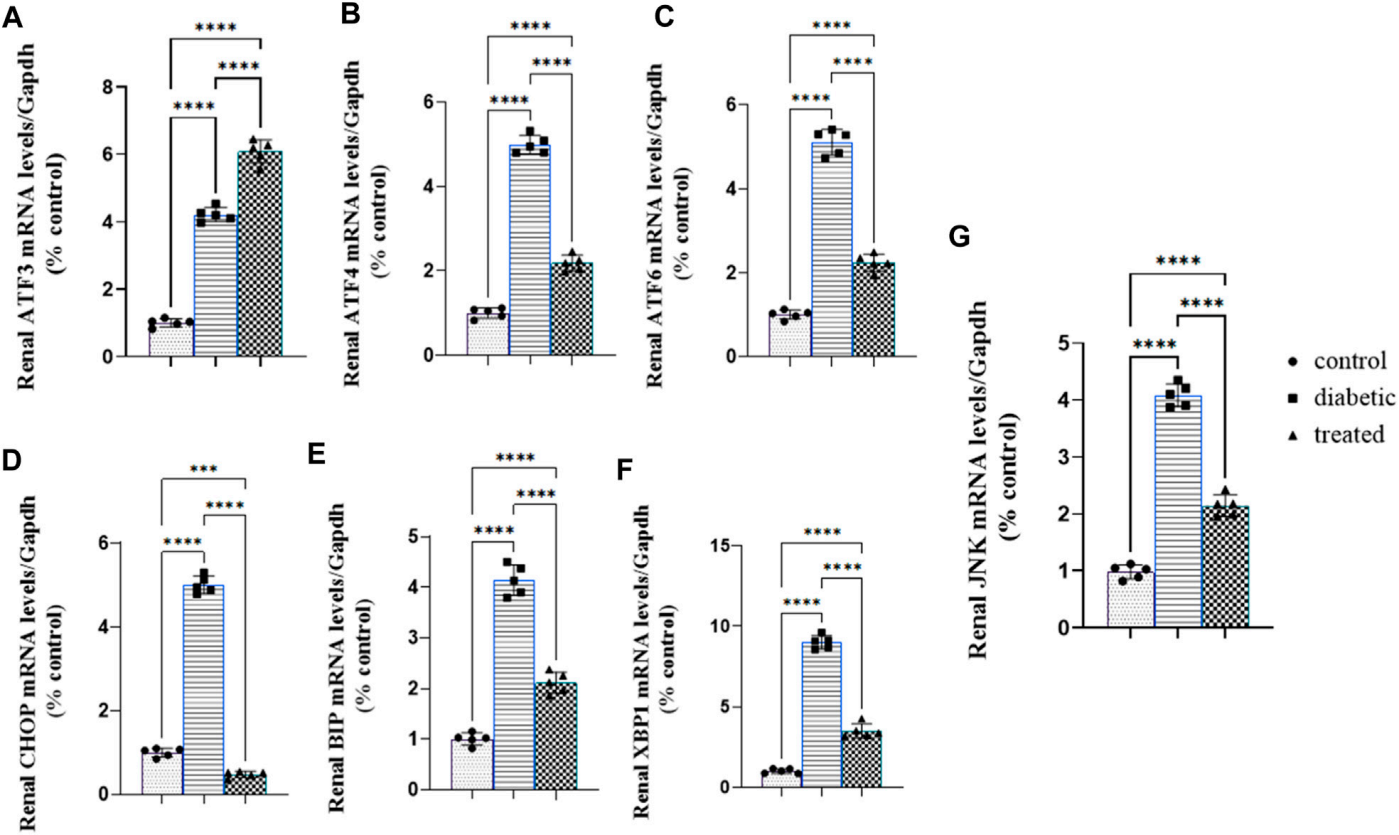
Effect of BM-MSC administration on the mRNA expression of renal ER stress markers **(A–G)**: **(A)** renal ATF3, **(B)** renal ATF4, **(C)** renal ATF6, **(D)** renal CHOP, **(E)** renal BIP, **(F)** renal XBP1, and **(G)** renal JNK. Values represent the mean ± SEM of 10 rats per group. *p < 0.05, **p < 0.01, ***p < 0.001, and ****p < 0.0001.

Type 1 diabetes induced a significant upregulation in the mean fold change of the relative mRNA expression of renal endoplasmic reticulum stress markers such as activating transcription factor 4 (ATF4), ATF6, ATF3, C/EBP homologous protein (CHOP), c-Jun amino-terminal kinases (JNK), X-box binding protein (XBP1), and immunoglobulin-binding protein (BIP) compared with the control group Figures 5A–G. Meanwhile, BM-MSC administration not only significantly downregulated the mean fold change of the relative mRNA expression of renal endoplasmic reticulum stress markers, ATF4, ATF6, CHOP, JNK, XBP1, and BIP, but also significantly upregulated the mean fold change of the relative mRNA expression of renal ATF3 compared with the diabetic group (27) Fig (26; A–G.).

### 3.6 Effect of BM-MSC transplantation on the mRNA expression of renal proapoptotic and antiapoptotic markers

**FIGURE 6:**
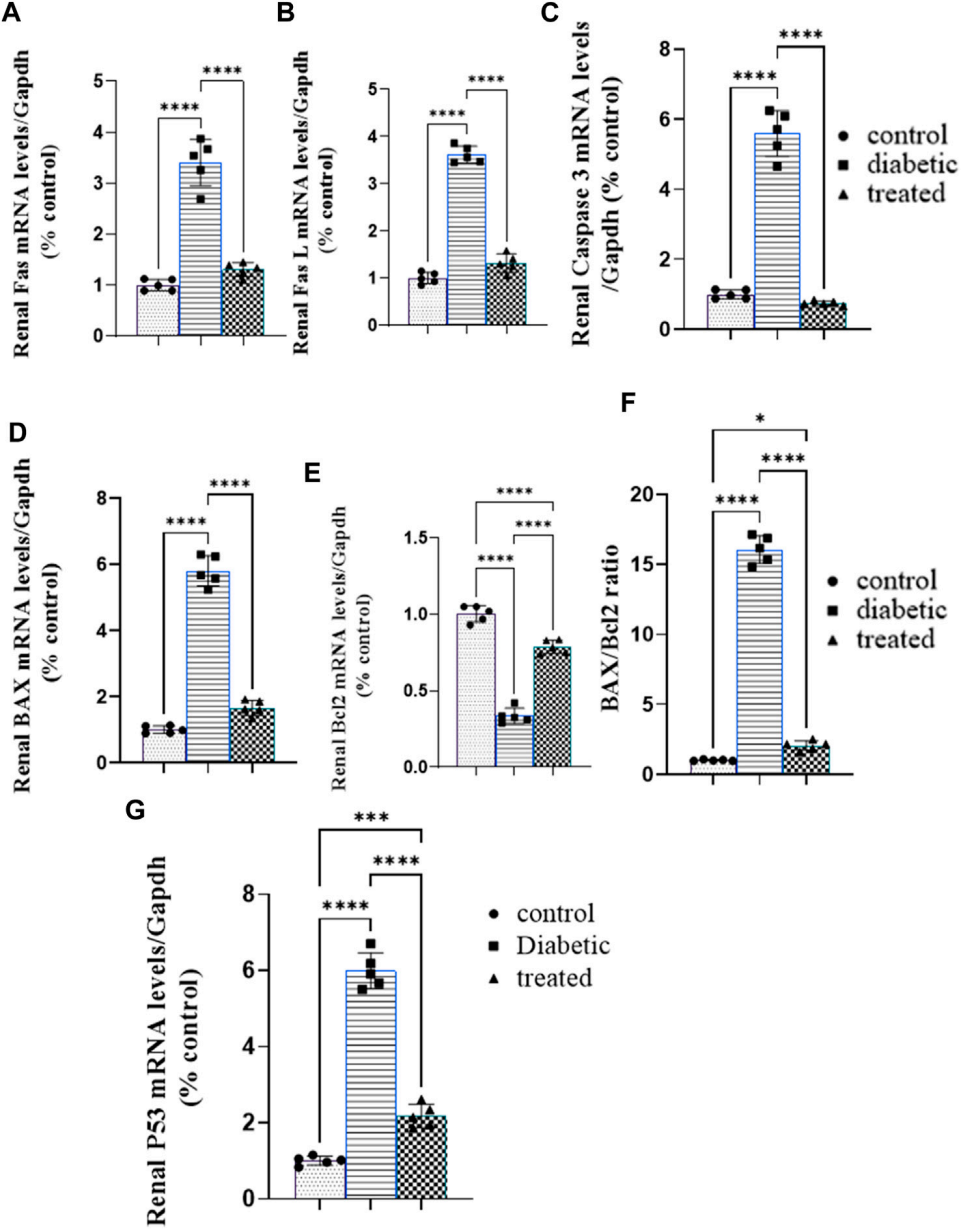
Effect of BM-MSC administration on the mRNA expression levels of renal proapoptotic and antiapoptotic markers **(A–G)**: **(A)** renal FAS, **(B)** renal FASL, **(C)** renal caspase-3, **(D)** renal BAX, **(E)** renal BCL2, **(F)** BAX/BCL2 ratio, and **(G)** renal P53. Values represent the mean ± SEM of 10 rats per group. *p < 0.05, **p < 0.01, ***p < 0.001, and ****p < 0.0001.

Type 1 diabetes induced a significant upregulation in the mean fold change of the relative mRNA expression of renal proapoptotic markers Fas, FasL, P53, caspase-3, BAX, and BAX/BCL2 and a significant downregulation in the antiapoptotic marker BCL2 compared with the control group (Figures 6A–G). BM-MSC administration significantly downregulated the mean fold change of the relative mRNA expression of renal proapoptotic markers Fas, FasL, P53, caspase-3, BAX, and BAX/BCL2 and significantly upregulated the antiapoptotic marker BCL2 compared with the diabetic group (Figures 6A–G).

### 3.7 Effect of BM-MSC transplantation on renal histopathological finding and immunohistochemical analysis

**FIGURE 7:**
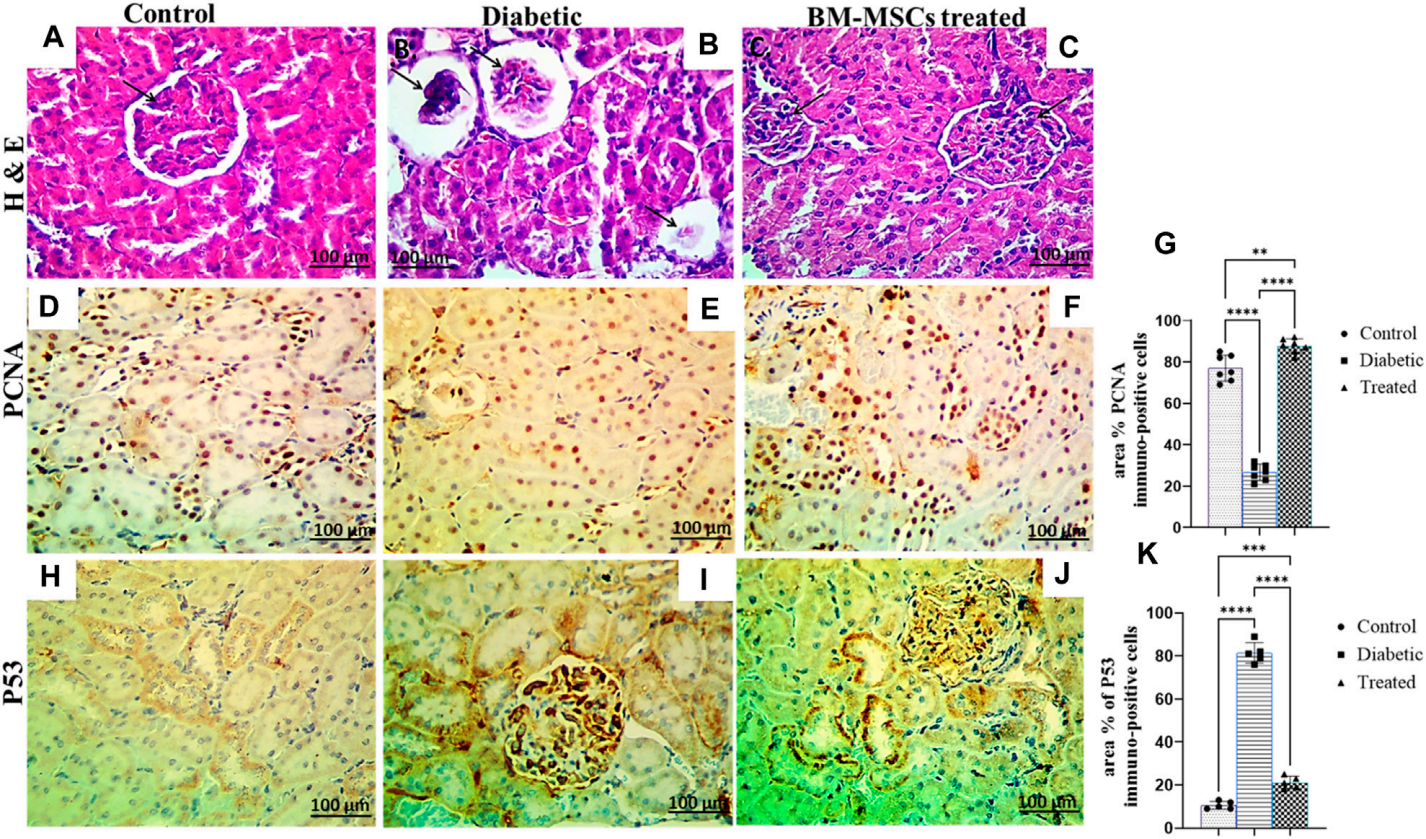
Effect of BM-MSC administration on renal histological characteristics (H&E-stained sections) and the immunohistochemical staining of PCNA and P53, as indicated by a +ve immune reaction **(A–K)**. **(A)** Photomicrograph of a renal cross-section stained by H&E (×400) shows a normal pattern of renal tissue in the control group, **(B)** photomicrograph of a renal cross-section stained by H&E (×400) shows collapsed and necrotic glomeruli with a widening of ‘Bowman’s space in the diabetic group, **(C)** photomicrograph of a renal cross-section stained by H&E (×400) shows a structured renal architecture in the BM-MSC-treated group compared to the normal control group, **(D)** photomicrograph of a renal cross-section immunostained with PCNA in the control group, **(E)** photomicrograph of a renal cross-section immunostained with PCNA in the diabetic group, **(F)** photomicrograph of a renal cross-section immunostained with PCNA in the BM-MSC-treated group, **(G)** PCNA immunostaining intensity (percent area), **(H)** photomicrograph of a renal cross-section immunostained with P53 in the control group, **(I)** photomicrograph of a renal cross-section immunostained with P53 in the diabetic group, **(J)** photomicrograph of a renal cross-section immunostained with P53 in the BM-MSC-treated group, and **(K)** P53 immunostaining intensity (percent area). Values represent mean ± SEM of 10 rats per group. *p < 0.05, **p < 0.01, ***p < 0.001, and ****p < 0.0001.

The histopathological examination of control rat kidney sections showed a normal histological picture of the renal glomeruli, and tubules (Figure 7A). However, the examination of the H&E-stained diabetic rat kidney revealed collapsed and necrotic glomeruli with a widening of ‘Bowman’s space (Figure 7B). On the other hand, the kidney of BM-MSC diabetic-treated rats showed an almost normal histological picture of the renal glomeruli and tubules (Figure 7C). Moreover, the result of the present investigation indicated a significant *p* < 0.001 downregulation in the positive immunostaining of the PCNA protein expression of the diabetic group compared to the control, which reverted with BM-MSC transplantation (Figures 7D–G) On the other hand, the findings of the current investigation revealed a significant *p* < 0.001 upregulation in the positive immunostaining of P53 of the diabetic rats compared with the control one; however, BM-MSC-treated groups showed significant downregulation in the positive immunostaining of P53 protein expression compared with the diabetic group (Figures 7H–J).

## 4 Discussion

Diabetic nephropathy is a diabetic complication affecting 40% of diabetic patients. DN is caused by several molecular processes including oxidative stress, ER stress, and inflammatory and apoptotic pathways (Zhuang et al., 2019). The current study’s findings validated the onset of diabetic nephropathy that was observed via an increase in serum urea, and creatinine levels, urine volume, urinary microalbumin, and albumin excretion and a decrease in urinary excretion of urea and creatinine. These results were consistent with the results previously obtained by Nobrega et al. (2004), Li et al. (2018), Zhao et al. (2018), Li et al. (2019), and Oraby et al. (2019). The aforementioned results can be attributed to the fact that hyperglycemia affected the function and vitality of both podocytes and tubular cells, as reported by Liu and Tang (2016), who found that hyperglycemia-induced apoptosis of podocytes was considered the key regulator for solute transportation from the tubulointerstitial compartment of the nephron; moreover, damaged podocytes were associated with proteinuria, renal fibrosis, and irreversible renal damage (Wolf et al., 2005). DN was accompanied by damage to podocytes that involved cellular hypertrophy, podocytopenia, and glomerulosclerosis (Liu and Tang, 2016). In addition, hyperglycemia damaged the proximal tubular epithelial cells (PTECs) accompanied by proteinuria, decreased renal function, tubulointerstitial fibrosis, and inflammation (Gilbert and Cooper, 1999).

On the contrary, BM-MSC administration showed that BM-MSCs can come to the injured renal tissue that is detected by tracking cells that were labeled with PKH-26 cell linker and decreasing serum levels of urea, creatinine, 24 h urine volume, and both urinary albumin and microalbumin excretion as well as increasing urinary urea and creatinine excretion. These findings were consistent with the following previously obtained results by Zhang et al. (2013), Lv et al. (2014), Li et al. (2018), Bai et al. (2019), and Wang et al. (2019). These results could be attributed to the regenerative capacity of BM-MSCs via secreting many growth factors (Kaingade et al., 2016) that stimulate the internal repairing mechanism via stimulating resident stem cells and soluble factors (Chen et al., 2007) that initiate a local anti-inflammatory microenvironment and prevent further destruction of both podocytes and proximal tubular epithelial cells reducing proteinuria, tubulointerstitial fibrosis, and glomerulosclerosis.

Furthermore, the current study’s findings showed a marked upregulation in the mRNA expression of desmin, vimentin, and nestin which were obtained by Zou et al. (2006), Sakairi et al. (2007), Su et al. (2007), and Liu et al. (2013). The aforementioned findings can be attributed to hyperglycemia-induced ER stress-induced inflammatory and oxidative stress, which is exacerbated by inflammatory cell infiltration and higher secretion of proinflammatory cytokines, which potentiate the precipitation of cytoskeleton protein and mesangial matrix protein (Lai et al., 2007). Interestingly, intraperitoneal administration of BM-MSCs significantly downregulated the expression of cytoskeleton proteins desmin, vimentin, and nestin, which was the same as that obtained by Liu and Tang (2016) and Kholia et al. (2018). Moreover, it was previously reported that the overexpression of desmin, vimentin, and nestin enhanced the migration of BM-MSCs to injured kidneys enhancing renal cellular regeneration (Wen et al., 2012). The aforementioned results could be attributed to the immunomodulatory function of BM-MSCs that downregulates proinflammatory cytokines, decreases renal infiltration with inflammatory cell, and interferes with inflammatory cell proliferation (Jiang et al., 2005; Krampera et al., 2006; Chen et al., 2007), which is considered the main cause for upregulating the expression of cytoskeleton proteins (Lai et al., 2007).

Regarding the oxidative stress caused by hyperglycemia in the kidney, the current investigation found a substantial increase in lipid peroxidation markers and a decrease in antioxidant capacity, which is consistent with the prior findings by Lee et al. (2003), Xu et al. (2017), Amaral et al. (2018), and Gong et al. (2019). The hyperglycemic state experienced with type 1 diabetes that leads to decreased mitochondrial membrane potential and increased generation of ROS and RNS that caused micro- and macrovascular alteration ended with DNA damage, the overexpression of extracellular matrix protein precipitation, mesangial expansion, glomerular fibrosis, and glomerular atrophy (Kashihara et al., 2010). However, BM-MSC administration ameliorated hyperglycemia-induced renal oxidative stress, decreasing lipid peroxidation markers and increasing ROS scavenging enzymes that go hand in hand with those previously obtained by Valle-Prieto and Conget (2010), Lv et al. (2014), Al-Rasheed et al. (2018), and Bai et al. (2019). It could be attributed to the lowering FBG effect of BM-MSCs illustrated in the current study’s findings that balanced the generation of ROS and oxidant scavenging system which ameliorated the micro/macrorenal vascular alteration that reduced the onset of glomerular sclerosis, atrophy, and mesangial expansion (Valle-Prieto and Conget, 2010) altogether secreting several growth (Kaingade et al., 2016) and soluble factors (Lai et al., 2007) that induced internal repair via stimulating resident stem cells and/or control BM-MSC transdifferentiation in tubular cells and podocytes (Khalilpourfarshbafi et al., 2017).

The current study’s findings confirmed the aforementioned events, as they showed that the ER stress markers ATF6, ATF4, ATF3, CHOP, JNK, BIP, and XBP1 were significantly upregulated, and these results followed the previously obtained results by Wang et al. (2017); Fan et al. (2017); Xu et al. (2017); Yum et al. (2017); Yang and Wu (2018); and Shibusawa et al. (2019). Furthermore, renal ER stress activation may be due to hyperglycemia, advanced glycation end products (AGE), and free fatty acids (FFAs), which contribute to ER Ca+2 depletion that induced ER stress and apoptosis that is ameliorated with an exogenous chaperone (Morse et al., 2010; Cao et al., 2014).

Moreover, in the activation of the three pathways of ER, the first pathway was (PERK/eIF2α/ATF4/CHOP) responsible for the stimulation of eukaryotic translation initiation factor 2 alpha (eIF2α) that block the translation of secretory protein to decrease ER protein load and stimulate translation of activating factor 4 that stimulated the transcription of (Chaperone) BIP to bind with the ER sensors if the cause of ER stress was solved; if not, chaperone translocates to the cytoplasmic membrane that activated inflammation; in this case, the increase in the expression of C/EBP homologous protein (CHOP) induced podocyte and tubular cell apoptosis and increased amino acid metabolism (Harding et al., 2000). The result of the present study reflected the activation of the PERK pathway with the marked upregulation in mRNA of CHOP, ATF4, and BIP. The second pathway is the so-called IRE1α/XBP1/JNK pathway. This pathway was responsible for shutting down and splicing of the mRNA that encodes secretory proteins in a process called regulated IRE1 α-dependent decay (RIDD) to decrease ER protein load and spliced mRNA of X box-binding protein 1 (XBP1) that activated the transcription of ER hemostatic factors as chaperone and endoplasmic reticulum-associated degradation (ERAD) components that degraded unfolded and misfolded proteins in an attempt to resolve endoplasmic reticulum stress; if this stress was unresolved, XBP1 upregulated the expression of c-Jun N-terminal kinase (JNK) that activated podocytes, tubular apoptosis, and inflammation (Hollien and Weissman, 2006; Oikawa et al., 2009). The result of the present investigation indicated the activation of the aforementioned pathway, which was reflected by the marked upregulation in the mRNA expression of chaperone BIP, XBP1, and JNK.

The third pathway was the ATF6 pathway. When activating transcription factor 6 (ATF6), sensed UPR became unbounded from BIP and translocated to the Golgi apparatus to be spliced into two transcriptional factors that immediately move to the nucleus to upregulate the expression of the ERAD component that degraded mRNA, misfolded and unfolded protein, and chaperone to inhibit ER stress sensors (PERK, IREα, and ATF6) if the causes of ER stress were resolved, as well as XBP1 to increase the expression of ER hemostatic factors and ERAD components (Haze et al., 1999). Interestingly, the current study’s findings showed typical activation of the ATF 6 pathway that was illustrated by sharp upregulation in the mRNA expression of ATF6, ATF3, and BIP. However, BM-MSC administration reverted the condition of ER stress to the normal physiological tone with marked downregulation for all ER stress except ATF3, which is consistent with the previously obtained results by Okasha et al. (2017), Li et al. (2019), and Liao et al. (2019). Meanwhile, this elevation in ATF3 is considered interesting; ATF3 has been previously reported to play a beneficial role in high-fat diet (HFD)-induced diabetes and pancreatic ß-cell dysfunction (Zmuda et al., 2010). Oxidative stress and hyperglycemia considered the main inducers for ER stress (Cunard, 2015), secretion of several growth factors (Kaingade et al., 2016). Renal inflammation is considered one of the most important pathways that increase renal damage associated with type 1 diabetes (Cunard, 2015). ATF-3 upregulation displayed a cardio-protective effect via downregulating the expression of proapoptotic protein P53 and its related pathways, which was validated in a previous study which reported that the transfection of rat cardiomyocytes with ATF-3-loaded adenovirus exerted a protective effect against doxorubicin-induced cardiac apoptosis (Nobori et al., 2002). This study strongly confirmed our findings, as the BM-MSC-treated diabetic rats elicited a significant upregulation in the renal expression of ATF-3, which was associated with the overexpression of the proapoptotic proteins. However, the protective effect of the upregulated ATF-3 in the diabetic group might be overwhelmed by renal oxidative stress and overexpressed CHOP and JNK that potentially initiated renal cell injury and apoptosis (Cunard, 2015). The current study’s findings showed that type 1 diabetes elicited a marked renal inflammatory condition manifested by sharp upregulation in proinflammatory cytokines NFKβ, TNFα, IL1β, IL6, and IL8; these results were consistent with the previously obtained results by Sha et al. (2017), Al Hroob et al. (2018), and Xu and Ren (2019). The renal inflammatory condition could be discussed as the induction of ER stress-mediated oxidative stress, hyperglycemia, FFA, and AGE that manifested by increasing the cellular load of unfolded and misfolded proteins that were recognized by the immune cells as foreign protein, increasing inflammatory cell infiltration and inflammatory cytokine secretion, depletion of ER ca+2 reserve, and increasing cytosolic calcium content leading to the dislocation of ER chaperone from the ER membrane to the cytoplasmic membrane that is considered a regulatory protein presented to immune cells inducing inflammatory response (Cunard, 2015; Delong et al., 2016). In addition, the reaction between these proteins and immune cells leads to the formation of a complex that activates the expression of damage-associated molecular patterns (DAMPs); the expression of these patterns is considered an indicator of podocytes and tubular cell death (Panaretakis et al., 2008; Zhang et al., 2010).

On the other hand, several studies supported that ER stress activated the expression of NFKβ (Hu et al., 2006). In addition, IRE1α/XBP1 is considered the main inducer of inflammation (Kamimura and Bevan, 2008; Osorio et al., 2014); on the other side, ER stress eventually upregulated IL6, IL8, NFKβ, TNFα, and MCP1 (Fougeray et al., 2011). Interestingly, BM-MSCs ameliorated renal inflammatory conditions that illustrated a sharp downregulation in proinflammatory cytokines NFKβ, TNFα, IL1β, IL6, and IL8 following the work of Abdel Aziz et al. (2014), Li Y. et al. (2018), Bai et al. (2019), and Lee et al. (2019). This could be attributed to the immunomodulatory function of BM-MSCs (Hashemian et al., 2015).

Regarding the apoptosis of podocytes and tubular cells, the current study’s findings showed that type 1 diabetes significantly upregulated the mRNA expression of proapoptotic markers FAS, FAS l, P53, BAX, and caspase-3 and downregulation in the antiapoptotic marker BCL2 that were in the same line with the previously obtained findings by Kumar et al. (2004), Sha et al. (2017), Gong et al. (2019), Ju et al. (2019), and Xu and Ren )2019).

Type 1 diabetes-induced renal cell apoptosis could be attributed to oxidative stress (Sha et al., 2017), endoplasmic reticulum stress (Erekat et al., 2019), and cytokine-mediated renal apoptosis (Sha et al., 2017) that activated the phosphorylation of P53 which increases the expression of BAX and decreases the expression of antiapoptotic factor BCL2; then, BAX-activated cytochrome C that activates cleavage of pro-caspase 9 into active caspase 9 and activated caspase 3 ended with the propagation of the apoptotic pathway (Tomita, 2017). So, it could be supposed that type 1 diabetic induced renal apoptosis in a pathway of (insulin-/hyperglycemia+/redox+/ER stress+/inflammatory cytokines+/FAS+/FAS L +/BAX +/caspase-3+/P53+/BCL2-). Surprisingly, BM-MSC administration ameliorated renal apoptosis noticed with a marked upregulation in antiapoptotic marker BCL 2 mRNA expression and downregulation in proapoptotic marker (FAS/FAS L/BAX/caspase-3/P53) mRNA expression, and these results were inconsistent with the results obtained by Abdel Aziz et al. (2014) and Ni et al. (2015) who reported that MSC administration ameliorated podocyte and tubular epithelium apoptosis. These findings can be attributed to BM-MSCs’ potential to ameliorate apoptosis inducers, either hyperglycemia or oxidative stress or beta-cell inflammatory environment or ER stress according to the result of the present work that reduced further renal tissue damage and apoptosis (Bugliani et al., 2019). In addition, these findings could be reverted to the elevated expression of activated transcription factor 3 (ATF3) that inhibits apoptosis via downregulating the expression of proapoptotic factor P21 (Hao et al., 2014) on the other hand. Altogether, BM-MSCs could ameliorate beta cell apoptosis in a molecular pathway (ATF3+/P21-/FAS-/FAS L -/BAX -/caspase-3-/P53-/BCL2+) or (insulin+/hyperglycemia-/redox-/ER stress-/inflammatory cytokines-/FAS-/FAS L -/BAX -/caspase-3-/P53-/BCL2+).

## 5 Conclusion

STZ-induced DN is a complex and multifactorial disease that involves various molecular mechanisms. Collectively, type 1 diabetes induces renal injury via inducing oxidative stress, ER stress, inflammatory condition, and apoptosis which could be reverted with the intraperitoneal administration of BM-MSCs. Such results provide important insights into the potential use of BM-MSCs as a novel therapeutic approach for DN. While MSCs hold great promise in the field of regenerative medicine, it is important to be aware of potential adverse effects that may arise from their use. Further studies are needed to confirm these findings and assess the long-term safety and efficacy of BM-MSC treatment in DN patients.

## Data Availability

All contributions are available in the article/Supplementary Material.
