# Corrigendum: BM-MSCs alleviate diabetic nephropathy in male rats by regulating ER stress, oxidative stress, inflammation, and apoptotic pathways

**DOI:** 10.3389/fphar.2024.1394151

**Published:** 2024-05-13

**Authors:** Tarek Khamis, Adel Abdelkhalek, Hussein Abdellatif, Nourelden Dwidar, Ahmed Said, Rama Ahmed, Kerolos Wagdy, Rowina Elgarhy, Rawan Eltahan, Hisham Mohamed, Eman Said Amer, Maria Hanna, Tarek Ragab, Abdallah Kishk, Judy Wael, Eyad Sarhan, Linda Saweres, Mohamed Reda, Sara Elkomy, Abdalah Mohamed, Abdullah Samy, Ateya Khafaga, Youliana Shaker, Hamdy Yehia, Asma Alanazi, Mohammed Alassiri, Emil Tîrziu, Iulia Maria Bucur, Ahmed H. Arisha

**Affiliations:** ^1^ Department of Pharmacology and Laboratory of Biotechnology, Faculty of Veterinary Medicine, Zagazig University, Zagazig, Egypt; ^2^ Faculty of Veterinary Medicine, Badr University in Cairo, Badr, Egypt; ^3^ Department of Human and Clinical Anatomy, College of Medicine and Health Sciences, Sultan Qaboos University, Muscat, Oman; ^4^ Anatomy and Embryology Department, Faculty of Medicine, Mansoura University, Mansoura, Egypt; ^5^ College of Medicine, King Saud Bin Abdulaziz University for Health Sciences (KSAU-HS), Riyadh, Saudi Arabia; ^6^ King Abdullah International Medical Research Center, Riyadh, Saudi Arabia; ^7^ Department of Basic Sciences, College of Science and Health Professions, King Saud Bin Abdulaziz University for Health Sciences (KSAU-HS), Riyadh, Saudi Arabia; ^8^ Department of Pathology and Laboratory Medicine, King Abdulaziz Medical City (KAMC), Ministry of the National Guard—Health Affairs, Riyadh, Saudi Arabia; ^9^ Department of Animal Production and Veterinary Public Health, Faculty of Veterinary Medicine, University of Life Sciences, “King Mihai I” from Timisoara [ULST], Timisoara, Romania; ^10^ Department of Animal Physiology and Biochemistry, Faculty of Veterinary Medicine, Badr University in Cairo, Badr, Egypt; ^11^ Department of Physiology, Laboratory of Biotechnology, Faculty of Veterinary Medicine, Zagazig University, Zagazig, Egypt

**Keywords:** diabetic nephropathy, mesenchymal stem cells, bone marrow-derived mesenchymal stem cells, diabetes, apoptosis, ER stress, inflammation, intermediate filament proteins

In the published article, there was an error in Figure 1 as published. In the published version of the manuscript, there was an unintentional duplication of Figure 1E, which resulted in Figure 1D being covered. This duplication occurred due to a technical error during the formatting process. The corrected Figure 1 and its caption appear below.

**FIGURE 1:**
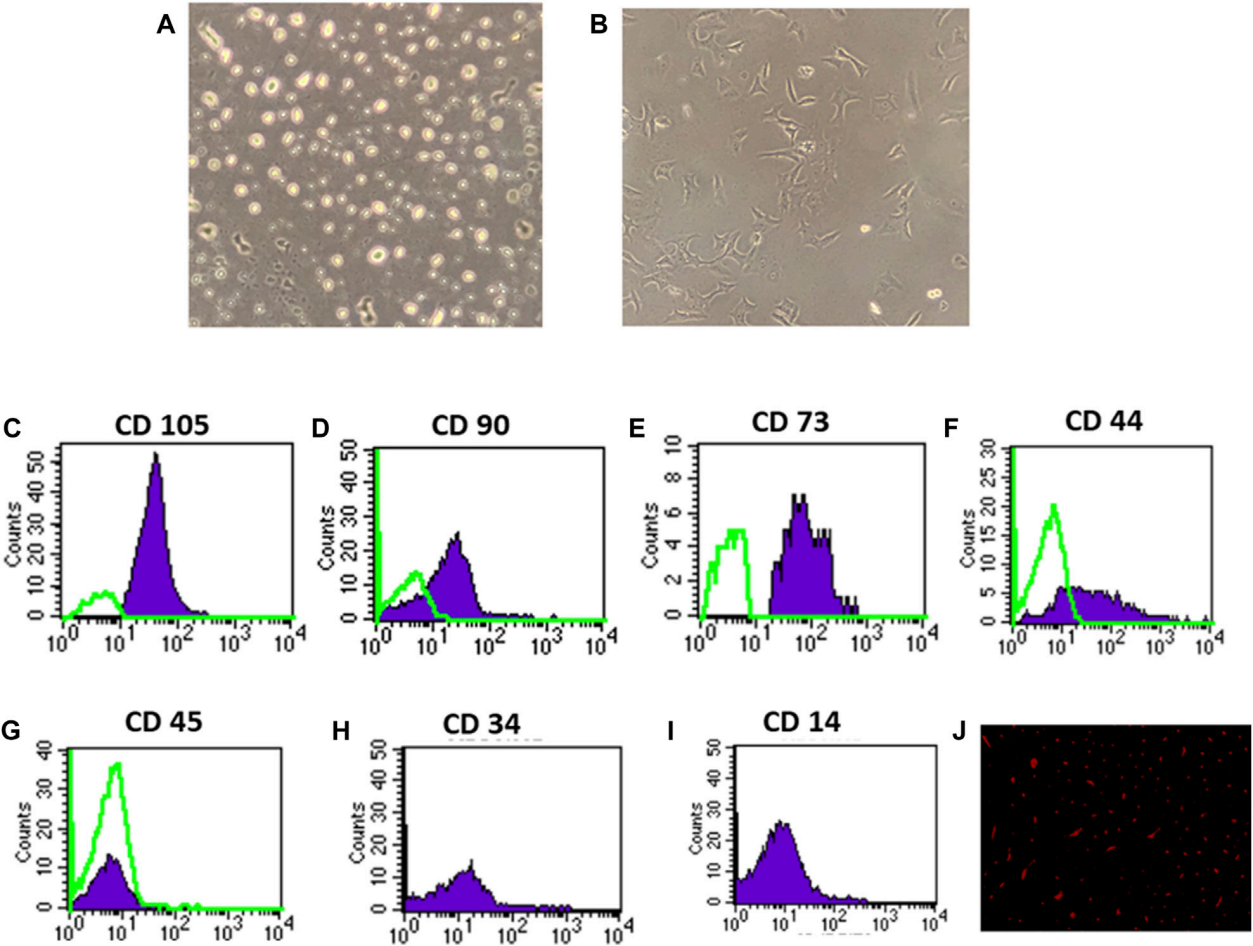
Identification and homing of BM-MSCs in diabetic rat renal tissue **(A–J)**. **(A)** BM-MSC isolation on the third day of culture; **(B)** BM-MSC isolation on the seventh day of culture **(C–I)**. Flow cytometric detection of BM-MSCs: **(C)** BM-MSC cell populations were +ve for CD105; **(D)** BM-MSCS cell populations were +ve for CD90; **(E)** BM-MSC cell populations were +ve for CD73; **(F)** BM-MSC cell populations were +ve for CD44; **(G)** BM-MSC cell populations were −ve for CD45; and **(H)** BM-MSC cell populations were −ve for CD34. **(I)** BM-MSC cell populations were −ve for CD14, and **(J)** PKH26 was used to identify BM-MSC homing in renal tissue.

In the published article, the **Supplementary Table S2** was mistakenly not included in the publication. The missing material contains the raw data of the article. This is now published alongside the original article.

The authors apologize for these errors and state that this does not change the scientific conclusions of the article in any way. The original article has been updated.

## Publisher’s note

All claims expressed in this article are solely those of the authors and do not necessarily represent those of their affiliated organizations, or those of the publisher, the editors and the reviewers. Any product that may be evaluated in this article, or claim that may be made by its manufacturer, is not guaranteed or endorsed by the publisher.

