# Supplementary material for: BM-MSCs alleviate diabetic nephropathy in male rats by regulating ER stress, oxidative stress, inflammation, and apoptotic pathways
Source: Front Pharmacol. 2023 Nov 16;14:1265230. doi: 10.3389/fphar.2023.1265230 (PMC10690373; doi:10.3389/fphar.2023.1265230)
Supplement: Supplementary file 2 [file Table1.docx]

BM-MSCs Alleviate Diabetic Nephropathy in Male Rats by Regulating ER Stress, Oxidative Stress, Inflammation, and Apoptotic Pathways

Tarek Khamis ^1,^* , Adel Abdelkhalek ^2^, Hussein Abdellatif^3, 4^, Nourelden Dwidar ^2^, Ahmed said ^2^, Rama Ahmed ^2^, kerolos Wagdy ^2^, Rowina elgarhy ^2^, Rawan eltahan ^2^, Hisham Mohamed ^2^, Eman said amer ^2^, Maria Hanna ^2^, Tarek Ragab ^2^, Abdallah Kishk ^2^, Judy Wael ^2^, Eyad Sarhan ^2^, Linda Saweres ^2^, Mohamed Reda ^2^, Sara Elkomy ^2^, Abdalah Mohamed ^2^, Abdullah Samy ^2^, Ateya Khafaga ^2^, Youliana Shaker ^2^, Hamdy Yehia ^2^, Asma Alanazi^5,6^, Mohammed Alassiri^6,7,8^, Emil Tîrziu ^9^, Iulia Maria Bucur ^9,^*, Ahmed Hamed Arisha ^10,11,^*

**Table 1.** Primer sequences for real-time PCR

| Gene | Forward primer sequence (5' to 3') | Reverse primer sequence (5' to 3') | Product size | Accession no. |
| --- | --- | --- | --- | --- |
| ATF3 | GCCATCCAGAACAAGCACC | ACTTGGCAGCAGCAATTT | **166** | **NM_012912.2** |
| ATF4 | CCTTCGACCAGTCGGGTTTG | CTGTCCCGGAAAAGGCATCC | 186 | NM_024403.2 |
| ATF6 | AAGTGAAGAACCATTACTTTATATC | TTTCTGCTGGCTATTTGT | 157 | NM_001107196.1 |
| Bax | CGAATTGGCGATGAACTGGA | CAAACATGTCAGCTGCCACAC | 109 | NM_017059.2 |
| Bcl-2 | GACTGAGTACCTGAACCGGCATC | CTGAGCAGCGTCTTCAGAGACA | 135 | NM_016993.1 |
| BIP | AACCAAGGATGCTGGCACTA | ATGACCCGCTGATCAAAGTC | 240 | NM_013083.2 |
| Casp-3 | GAGACAGACAGTGGAACTGACGATG | GGCGCAAAGTGACTGGATGA | 147 | NM_012922.2 |
| CHOP | CACAAGCACCTCCCAAAG | CCTGCTCCTTCTCCTTCAT | 158 | NM_001109986.1 |
| Desmin | CATTGAGACCCGGGATGGAG | AAAGCGACTGGGTGTGACAT | 146 | NM_022531.1 |
| Fas | GAGCGTTCGTGAAACCGACA | AGGTTGGTGCACCTCCACTTG | 128 | NM_139194.2 |
| FasL | CACCAACCACAGCCTTAGAGTATCA | CACTCCAGAGATCAAAGCAGTTCC | 172 | NM_012908.1 |
| Gapdh | GGCACAGTCAAGGCTGAGAATG | ATGGTGGTGAAGACGCCAGTA | 143 | NM_017008.4 |
| IL1β | CACCTCTCAAGCAGAGCACAGA | ACGGGTTCCATGGTGAAGTC | 81 | NM_031512.2 |
| IL6 | ATATGTTCTCAGGGAGATCTTGGAA | GTGCATCATCGCTGTTCATACA | 80 | NM_012589.2 |
| JNK | AGTGTAGAGTGGATGCATGA | ATGTGCTTCCTGTGGTTTAC | 182 | NM_053829.2 |
| Nestin | GGTAGGGCTAGAGGACCCAA | TGGGCAATTCAAGGATCCCC | 151 | NM_031140.1 |
| NF-𝜅B | CAGGACCAGGAACAGTTCGAA | CCAGGTTCTGGAAGCTATGGAT | 150 | NM_199267.2 |
| P53 | CATGAGCGTTGCTCTGATGGT | GATTTCCTTCCACCCGGATAA | 67 | [NM_030989.3](https://www.ncbi.nlm.nih.gov/entrez/viewer.fcgi?db=nucleotide&id=189083685) |
| TNF-α | AGGGTCTGGGCCATAGAAC | CCACCACGCTCTTCTGTCTAC | 103 | NM_012675.3 |
| Vimentin | GCACGTCTTGACCTTGAACG | TGAGGTCAGGCTTGGAAACG | 197 | NM_017008.4 |
| XBP1 | TTACGAGAGAAAACTCATGGGC | GGGTCCAACTTGTCCAGAATGC | 289 | NM_001004210.2 |
